# Newly regenerated axons via scaffolds promote sub-lesional reorganization and motor recovery with epidural electrical stimulation

**DOI:** 10.1038/s41536-021-00176-6

**Published:** 2021-10-20

**Authors:** Ahad M. Siddiqui, Riazul Islam, Carlos A. Cuellar, Jodi L. Silvernail, Bruce Knudsen, Dallece E. Curley, Tammy Strickland, Emilee Manske, Parita T. Suwan, Timur Latypov, Nafis Akhmetov, Shuya Zhang, Priska Summer, Jarred J. Nesbitt, Bingkun K. Chen, Peter J. Grahn, Nicolas N. Madigan, Michael J. Yaszemski, Anthony J. Windebank, Igor A. Lavrov

**Affiliations:** 1 Department of Neurology, Mayo Clinic, Rochester, MN USA; 2 School of Sport Sciences, Universidad Anáhuac México, Campus Norte, Huixquilucan, State of Mexico Mexico; 3 Department of Neurologic Surgery, Mayo Clinic, Rochester, MN USA; 4 Department of Neuroscience, Brown University, Providence, Rhode Island USA; 5 National University of Ireland Gallway, Gallway, Ireland; 6 Department of Neuroscience, Scripps College, Claremont, CA USA; 7 Paracelsus Medical Private University, Salzburg, Austria; 8 Division of Brain, Imaging, and Behaviour – Systems Neuroscience, Krembil Research Institute, Toronto Western Hospital, University Health Network, Toronto, ON Canada; 9 Institute of Medical Science, Faculty of Medicine, University of Toronto, Toronto, ON Canada; 10 Institute of Fundamental Medicine and Biology, Kazan Federal University, Kazan, Russia; 11 Department of Physical Medicine and Rehabilitation, Mayo Clinic, Rochester, MN USA; 12 Department of Orthopedic Surgery, Mayo Clinic, Rochester, MN USA; 13 Department of Biomedical Engineering, Mayo Clinic, Rochester, MN USA

**Keywords:** Spinal cord injury, Biomaterials - cells, Regenerative medicine, Drug delivery, Tissue engineering

## Abstract

Here, we report the effect of newly regenerated axons via scaffolds on reorganization of spinal circuitry and restoration of motor functions with epidural electrical stimulation (EES). Motor recovery was evaluated for 7 weeks after spinal transection and following implantation with scaffolds seeded with neurotrophin producing Schwann cell and with rapamycin microspheres. Combined treatment with scaffolds and EES-enabled stepping led to functional improvement compared to groups with scaffold or EES, although, the number of axons across scaffolds was not different between groups. Re-transection through the scaffold at week 6 reduced EES-enabled stepping, still demonstrating better performance compared to the other groups. Greater synaptic reorganization in the presence of regenerated axons was found in group with combined therapy. These findings suggest that newly regenerated axons through cell-containing scaffolds with EES-enabled motor training reorganize the sub-lesional circuitry improving motor recovery, demonstrating that neuroregenerative and neuromodulatory therapies cumulatively enhancing motor function after complete SCI.

## Introduction

Spinal cord injury (SCI) induced cell death, demyelination, and axonal damage leads to loss of independence of daily life. The spinal cord has limited ability to regenerate and currently available therapies including pharmacologic, cellular, and biomaterial agents demonstrated only modest functional recovery. Recent advances in bioelectronics and tissue engineering led to the rise of neuromodulatory and neuroregenerative therapies, which aim to target circuits in the brain or spinal cord to improve neurologic functions. Encouraging results using epidural electrical stimulation (EES; definition of major terms can be found in the glossary in the supplementary information) to restore motor function in humans with SCI^1–3^ have been attributed to the presence of functionally silent fibers and the combination of EES with motor training^4–6^. Most of the patients diagnosed with complete loss of motor control after SCI demonstrate some degree of sub-functional connectivity, which provides the anatomical and functional basis for supraspinal control in the presence of EES^6,7^. Currently, there is only limited understanding of the mechanisms underlying the effect of EES to restore motor functions after SCI. In this regard, elucidating the mechanisms of EES-enabled improvement in motor control in animal models with limited connectivity will advance EES therapy in SCI subjects and open the possibility of combining EES with neuroregenerative therapies.

According to the current concept, electrical stimulation engages the lumbosacral circuitry and can promote standing and stepping behavior in weight supported mice, rats, and cats without supraspinal input after complete transection^8–13^. The type of movement elicited depends on the level of the spinal cord stimulation, however, the optimal location for step like movement was found at L2 and S1 spinal segments that was applied in monopolar^10^ and bipolar configurations^11,14–17^ improving stepping in complete SCI rats. Another important component of EES-enabled stepping is activity-dependent plasticity of the sensorimotor circuitry induced by intensive motor training^8,12,15,16,18–23^. EES-enabled motor training was able to induce overground walking, stairclimbing, and swimming ability in rats with severe SCI^16,24^. The motor cortex is likely involved in the recovery of these behaviors though the development of neuronal detours in the brainstem, supralesional spinal neurons, or with intact neuronal tissues across the lesion^16,24^. In addition, trained rats have less variability in electromyogram (EMG) and greater localized c-fos immunoreactivity in the lumbosacral spinal cord, suggesting the task-dependent plasticity^21^. Together these findings demonstrated that motor control and improved gait could be achieved through regeneration of the short spinal tracts, formation of detours, and plasticity of neural circuits without necessarily regeneration of long descending tracts^25^.

One of the reasons for poor regeneration after SCI is the formation of the cyst and scar tissue rich with inhibitory molecules that prevent axons from crossing the injury site. Neuroregenerative therapies aim to promote regrowth through cell replacement, microenvironment modifications, tissue engineering, and promotion of intrinsic and extrinsic growth pathways. One tissue engineering approach is to utilize biomaterials to bridge the lesion following SCI. Polymer scaffolds provide physical guidance and structure for axonal growth through the injury. When implanted alone, they have been found to reduce scar and cyst formation^26–29^. Scaffolds combined with other factors to promote tissue growth and vascularization, as well as delivery drugs and cells, demonstrated further improvement in multiple models^30–35^. Xu et al.^36^ found that loading guidance tubes with Matrigel and Schwann cells significantly improved axon regeneration following spinal cord transection. The delivery of brain derived growth factor (BDNF) and neurotrophin-3 (NT-3) through minipumps along with Schwann cell loaded guidance tubes further enhanced axonal regeneration^36^. Our group has constructed a multichannel scaffold containing positively charged oligo [poly(ethylene glycol)fumarate] (OPF+). This scaffold has biomechanics similar to the rat spinal cord and the positive charge aids in enhancing cell attachment and axonal outgrowth^27,37^. When compared to poly (lactic co-glycolic acid) (PLGA) and poly(ɛ-caprolactone fumarate) (PCLF), OPF+ had greater number of axons contained in the channels^27^. OPF+ scaffolds loaded with Schwann cells overexpressing glial derived growth factor (GDNF) demonstrated enhanced axonal regeneration and myelination associated with recovery in hind-limb movements^38^. Another important finding of this study is that the regenerating axons were primarily ascending sensory axons located up to 30 mm away from the lesion. The OPF+ biomaterial could be further modified to contain drug eluting microspheres loaded with small molecule such as the anti-fibrotic drug rapamycin, demonstrating functional improvement that could be attributed to reduced fibrotic scarring^39^. These studies demonstrate that tissue engineering approaches are ideal for use in combinatorial treatments and that regeneration of spinal tracts can be promoted through the injury site, enabling anatomical reconnections that can be further functionally engaged with neuromodulation.

Recent advances in bioelectronics and tissue engineering demonstrate that neuromodulatory and neuroregenerative therapies independently can lead to functional restoration after SCI^40–44^. The combination of the different therapies considered to be critical for treatment of SCI due to multifaceted pathophysiological response to SCI. Recently reported combinatorial approach used in a phase I clinical trial utilized collagen scaffolds (NeuroRegen scaffold, InVivo Therapetuics) loaded with human umbilical cord mesenchymal stem cells implanted into patients with complete SCI (C5-T12) following surgical scar resection^45^. This study revealed no adverse effects and limited improvements in sensation level, motor evoked potentials, trunk stability, finger activity, and autonomic recovery in some patients 1 year after implantation. Our previous preclinical work demonstrated that axon regeneration was greater when Schwann cell loaded OPF+ scaffolds were used when compared to neural stem cells or mesenchymal stromal cells loaded scaffolds^46,47^. Regardless, most of the animal studies and clinical trials with cell therapies only demonstrated a modest functional recovery^48–51^. Spinal cord neuromodulation was also broadly studied in animal models and was successfully implemented in clinical trials, demonstrating restoration of neurologic functions in patients with clinically complete SCI^1,2,52–54^. Currently, there is little to no evidence that EES of the lumbroscaral spinal cord could promote axon regeneration alone, however, it likely exerts some neuroplasticity effect through increased excitability and plasticity of the local circuitry, as well as by utilizing the remaining connections that may exist following a clinically complete injury^25,51^. Both biomaterial or cell therapy and neuromodulation therapy being advanced to the clinical trials, however, up to know never been tested for their synergistic effect in preclinical studies on complete SCI models. Particularly, great potential for the combination of neuroregenerative and neuromodulation therapies represent a model with complete spinal transection, and with scaffold placement that could inform a better understanding of the role of sub-functional connectivity via newly regenerating across injury fibers in enabling neurologic functions with EES.

Elucidating the mechanisms of EES-enabled restoration of motor function after SCI with sub-functional connectivity is critical for optimizing combined neurostimulation and neuroregenerative therapy. To this end, we have developed an approach with hydrogel scaffold loaded with cells and small molecules used to enhance regeneration combined with electrodes that deliver EES-enabled rehabilitation. This approach provides application of biotechnologies currently being investigated in preclinical and clinical trials separately^1,45,52,53^. The positively charged OPF would help bridge the injury and provide scaffolding for axons^55–58^, rapamycin is well known for its ability to reduce fibrosis and microglial activation^39,59–61^, Schwann cells would help to remyelinate and provide some trophic support^62–64^, whereas GDNF would help promote axon regeneration and neuroprotection^38,65–68^. The synergistic effect of neuroregenerative therapy applied with EES-enabled motor training provides a strong platform to further enhance recovery through the selective regeneration of new fibers, neuroplasticity, and activation of the spinal circuitry involved in functional restoration. In this study, we report evidence of the contribution of newly regenerated axons through a scaffold in reorganization of the spinal circuitry and restoration of EES-enabled motor functions. The results of this work highlight the important interplay between neuroregenerative and neuromodulatory therapies in anatomical and functional restoration following SCI.

## Results

### Axon number across scaffolds does not differ among experimental groups

**Fig. 1:**
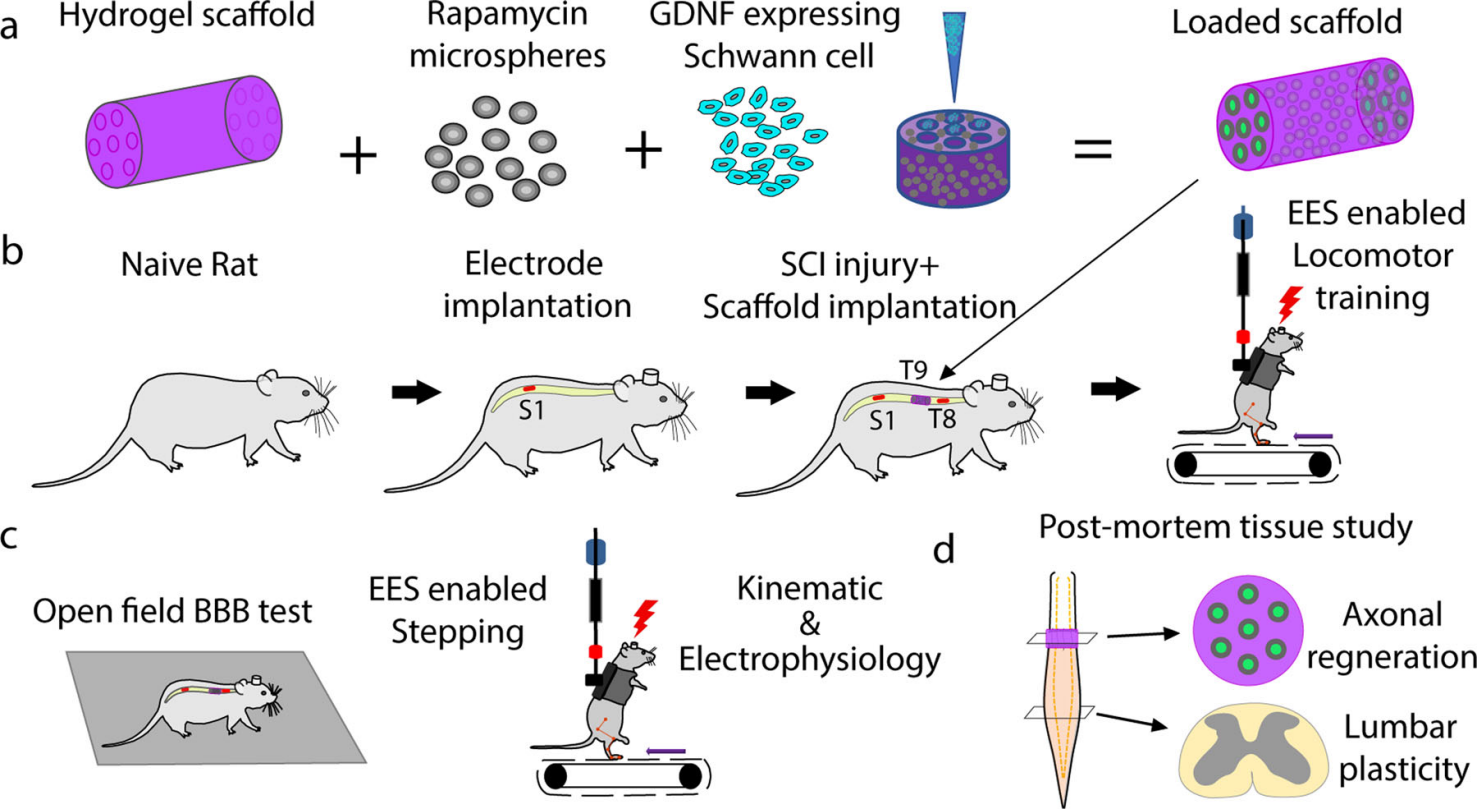
Experimental methods and study design. **a** Preparation of 7 channel OPF+ hydrogel scaffold containing rapamycin microspheres and channels loaded with GDNF expressing Schwann cells. **b** In vivo experimental model with implantation of the electrodes and scaffold following complete transection (T9). There were three groups of rats that received a T9 transection with epidural electrical stimulation (EES only), T9 transection with scaffold placement (scaffold only), and transection with scaffold placement and EES (combined therapy). **c** Following SCI and implantations, all rats received EES-enabled motor training on a treadmill with outcome collected as open field BBB score, kinematic, and electrophysiology during EES-enabled stepping on treadmill. **d** At the end of the experiment, axonal regeneration through the scaffold and change in plasticity across lumbosacral spinal cord were assessed.

The transplantation of scaffolds with GDNF secreting Schwann cells^38^ or with rapamycin and Schwann cells^39^ have shown functional and anatomical improvement after SCI. As an experimental platform for this study, we combined hydrogel scaffolds composed of positively charged oligo-[poly(ethylene glycol)fumarate] (OPF+) loaded with neurotrophic factor (GDNF) secreting Schwann cells and rapamycin microspheres (enhanced scaffolds) combined with EES and EES-enabled motor training (Fig. 1a–c)^13,69–72^. Groups with SCI and EES-facilitated training (*EES only*) (*n* = 4), with enhanced scaffolds *(scaffold only)* (*n* = 3), and with *combined therapy* of scaffold and EES-enabled training (*n* = 10, 5 with re-transection) were compared (Fig. 1b). At 7 weeks after complete SCI, the number of axons passing through each of the seven channels of the scaffold was assessed (Fig. 1d–g and Supplementary Fig 1).

**Fig. 2:**
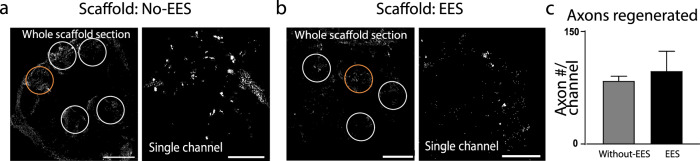
Axon regeneration through implanted scaffolds 7 weeks after injury. **a** Transverse section (10 µm thick) through a scaffold implanted in a rat that did not receive EES and **b** transverse section in rat that received EES-enabled motor training. The sections were fixed and stained with β-tubulin 7 weeks of post-implantation and axons were visualized and quantified in the channels (white circles) (scale bar = 400 μm. The channels in the orange circles are presented in higher magnification on the right (scale bar = 50 μm). **c** The number of axons passing through the scaffold shows no statistical difference between group without EES (*n* = 3) and group that received EES-enabled motor training (*n* = 5) (*t*-test). Error bars: +/− standard error of the mean (SEM).

Groups with scaffold only (Fig. 2a) and with combined therapy (Fig. 2b) demonstrated regeneration through the scaffold, although, with no difference between the groups: 84.46 ± 5.93 and 97.60 ± 26.16 axons/channel, respectively (Fig. 2c). There was also no statistical difference between the axon numbers in the dorsal, central, or ventral channels nor rostral to caudal quarter lengths (Supplementary Fig. 1). Approximately, equal number of axons are present through the length of the scaffold.

### Motor performance is enhanced with combined therapy

**Fig. 3:**
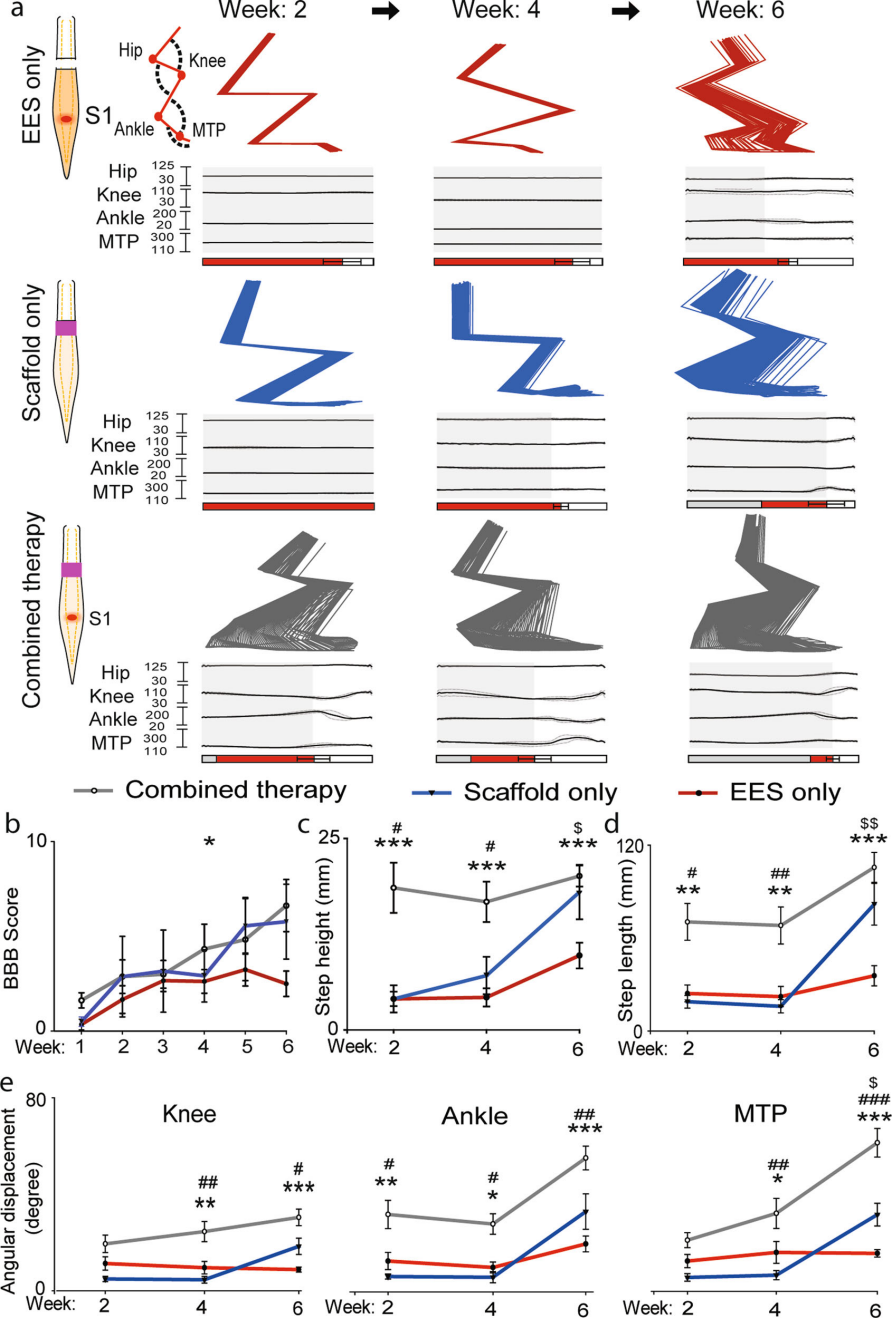
Rats receiving combined therapy showed early and sustained recovery. **a** Three representative examples of EES-enabled gait function recovery in animals after complete transection (T9) receiving EES only, Scaffold only or Combined therapy, collected at up to 6 weeks. Examples of stick diagram and joint angles were averaged from five consecutive steps on treadmill. Retro-reflective markers were placed on bony landmarks at the iliac crest, greater trochanter, lateral condyle of the femur, lateral malleolus and the distal end of the fifth metatarsal on both legs of the rat and were recorded to assess the kinematics of the hip, knee, ankle and MTP joints. Stance phase is represented by gray rectangles, drag by red rectangles, and swing by white rectangles. **b** Motor performance assessed with BBB score in rats with EES only (*n* = 4), with Scaffold only (*n* = 3), and with Combined therapy (*n* = 5) was accessed for 6 weeks of post-injury (*p < 0.05, two-way ANOVA with Tukey’s multiple comparisons). Gait kinematic parameters, step height (**c**), and step length (**d**), were compared between the groups at 2, 4, and 6 weeks after spinal cord transection. At each time points these parameters were found to be significantly improved in rats with Combined therapy, compared to the rats with EES only. Displacement of joint angles, knee, ankle, and MTP (**e**) (*p < 0.05, **p < 0.01, and ***p < 0.001, one-way ANOVA and post hoc analysis using Holm-Sidak method. Outcome presented EES only vs. Combined therapy^*^, with Scaffold only vs. with Combined therapy^#^, with EES only vs. with Scaffold only^$^). Error bars: +/− standard error of the mean (SEM).

**Fig. 4:**
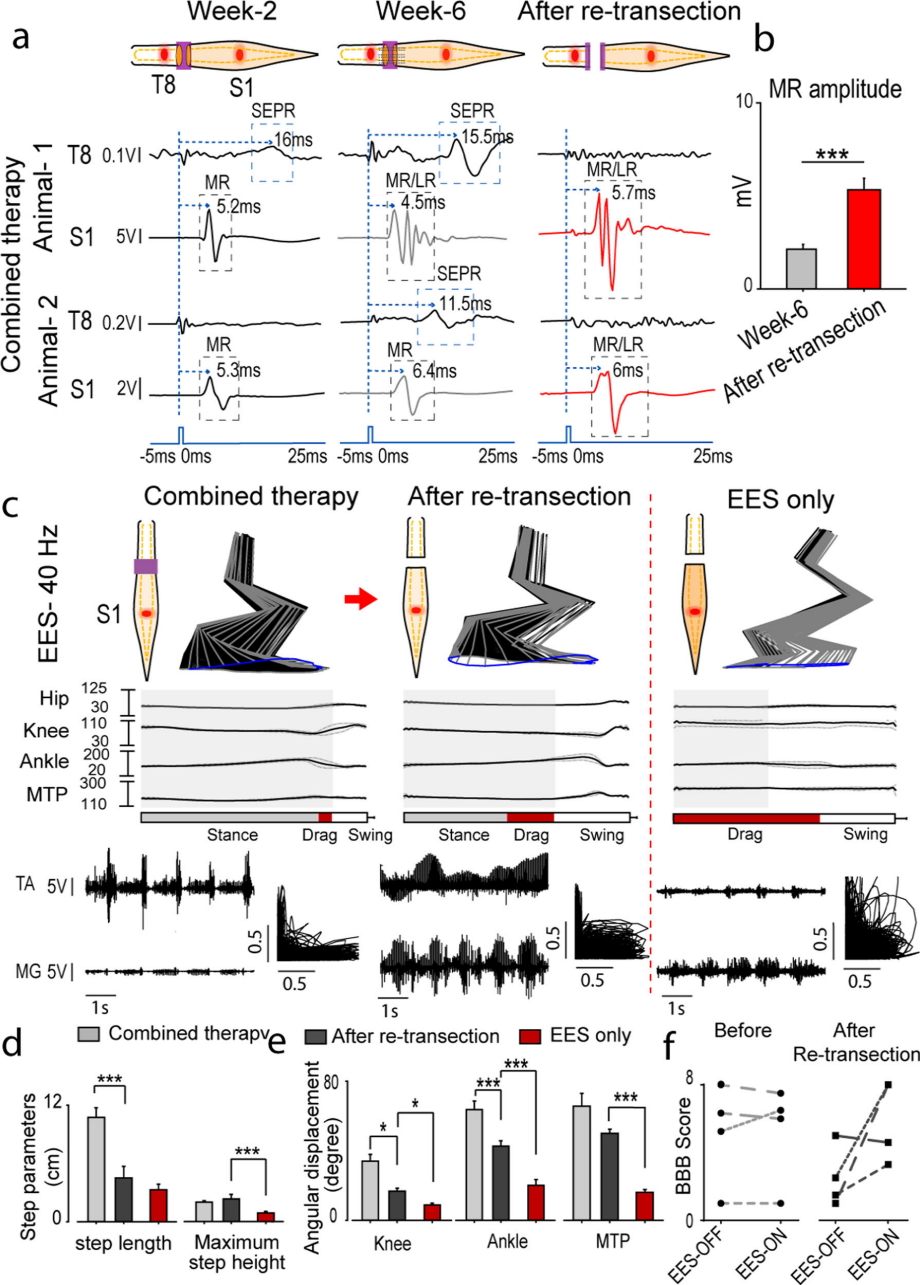
Electrophysiological and behavioral outcomes after re-transection of the scaffold compared to EES only. **a** Representative example of averaged spinal cord motor evoked potential (SCMEP) and supralesion evoked polysynaptic response (SEPR) (*n* = 8 responses) in rats implanted with hydrogel scaffold, and received EES-enabled motor training, collected at 2 and 6 weeks after spinal transection and after re-transection across the scaffold following 6 weeks of recovery. Motor evoked potentials were recorded from hind-limb muscle medial gastrocnemius (MG), while spinal cord was stimulated using epidural electrode placed at T8 segment (above the injury, SEPR) and at S1 segment (below the injury, SCMEP). Examples were collected from representative animal-1 and animal-2. Blue dotted line indicates the moment when EES pulse was applied. Middle and late responses (MR/LR) and SEPR are indicated with gray and blue rectangles. **b** Peak-to-peak amplitude of MR at week 6 and after re-transection. **c** Animals with scaffolds and EES (Combined therapy) recovered greater angular displacements of the knee, ankle, and MTP. After re-transection at week 6, improvement in motor function was still greater compared to transected animals without scaffold (EES only). The stance phase is represented by gray rectangle, drag by red, and swing by white. **d** Comparison of gait parameters, step length and maximum step height. **e** Angular displacements of hip, knee, ankle, and MTP. **f** BBB score assessed with EES OFF and ON before and after re-transection (*n* = 4). The same rats are tracked before and after re-transection using same types of dashed line. (Data are represented as mean +/− standard error. *p < 0.05, **p < 0.01, and ***p < 0.001, one-way ANOVA and post-hoc analysis using Holm-Sidak method). Error bars: +/− standard error of the mean (SEM).

EES-enabled motor performance was evaluated with kinematics (Fig. 3), EMG, and electrophysiology (Fig. 4). Open field behavior *Basso, Beattie, and Bresnahan (BBB) motor scores* were collected with and without EES. BBB scores at week 1 without stimulation were similar across all groups (0–1.5) with no difference during the first four weeks. At week 4 and 6, the BBB score was greater for rats with combined therapy (week 6: 6.62 ± 1.38) compared to EES only (week 6: 2.50 ± 0.67, *p* < 0.05; Fig. 3b). *Kinematic analysis* (Fig. 3a–e) at 2–4 weeks demonstrated higher step height in the group with combined therapy compared to animals with scaffold only (*p* < 0.05) and with EES only (*p* < 0.001). At week 6 the group with scaffolds only recovered step height almost to the level of group with combined therapy. Both groups with scaffold only and with combined therapy demonstrated higher step height compared to EES only (*p* < 0.05) (Fig. 3c). An increase in step length was found in the group with combined therapy at 2–4 weeks compared to scaffold only (week 2: *p* < 0.05, week 4: *p* < 0.01) or with EES only (week 2 and 4: *p* < 0.01; Fig. 3d). At week 6, the group with scaffolds only recovered to the level of the group with combined therapy. At 6 weeks after SCI, dragging was greater in the group with EES only compared to both combined therapy (*p* < 0.001) and scaffold only (*p* < 0.05) (Fig. 3a). All groups demonstrated recovery of angular displacement that in knee was greater at week 4 and 6 in group with combined therapy compared to other groups (*p* < 0.05). Metatarsophalangeal (MTP) joint angles were not different at 2 weeks after injury, although at 4–6 weeks, the group with combined therapy demonstrated greater MTP displacement compared to other groups (*p* < 0.05) (Fig. 3e).

### Electrophysiological evidence of functional regeneration across the scaffold

*Spinal cord motor evoked potentials (SCMEP)*^73^ were recorded while rats were stimulated above (T8) and below (S1) the injury at week 2 and 6, and after re-transection at week 7 (Fig. 4a). In a representative rat with combined therapy, stimulation above the injury (T8) at week 2 evoked small supralesional evoked polysynaptic response (SEPR) (Fig. 4a). By week 6 SEPR became more prominent and disappeared after re-transection. Stimulation below the injury at S1 evoked middle response (MR) (4.5–5.7 ms) that increased in amplitude following re-transection, indicating the influence of regenerated fibers on the sub-lesional network. In another rat, with stimulation at T8, SEPR was detected only at week 6 and also disappeared after re-transection (Fig. 4a). Stimulation below the injury at S1 in this rat also demonstrated facilitation of MR response after re-transection. The mean MR peak-to-peak amplitude before re-transection at week 6 was 4.28 mV and increased to 10.67 mV after re-transection (*p* < 0.001) (Fig. 4b).

*Electrophysiological and behavioral features* of motor performance were compared during EES-enabled motor training at 6 weeks after SCI and after re-transection. Rats with combined therapy demonstrated gradual recovery of EES-enabled stepping and after re-transection they retained some improvements compared to EES only group, suggesting influence of regenerated axons on reorganization of sub-lesional circuitry (Fig. 4c). Following re-transection, rats with combined therapy lost step length (*p* < 0.001) (Fig. 4d) as well as had a decrease in knee and ankle angles that still were greater compared to EES only group (*p* < 0.05) (Fig. 4e). The MTP angle did not change after re-transection, although was greater (*p* < 0.001) compared to EES only group (Fig. 4e). Interestingly, rats with combined therapy demonstrated similar BBB scores with EES turned OFF and ON, although, after re-transection, BBB score with EES-ON at optimal intensity for motor performance was increased for three of the four rats compared to when the EES was completely OFF. This may indicate on predominant role of trans-lesional connectivity on motor performance in case of partial connectivity. These results also suggest more evident role of EES on motor performance after complete spinal cord injury compared to partial connectivity.

### Regenerating axons influence synaptic reorganization of L2-S1 cord with combined therapy

**Fig. 5:**
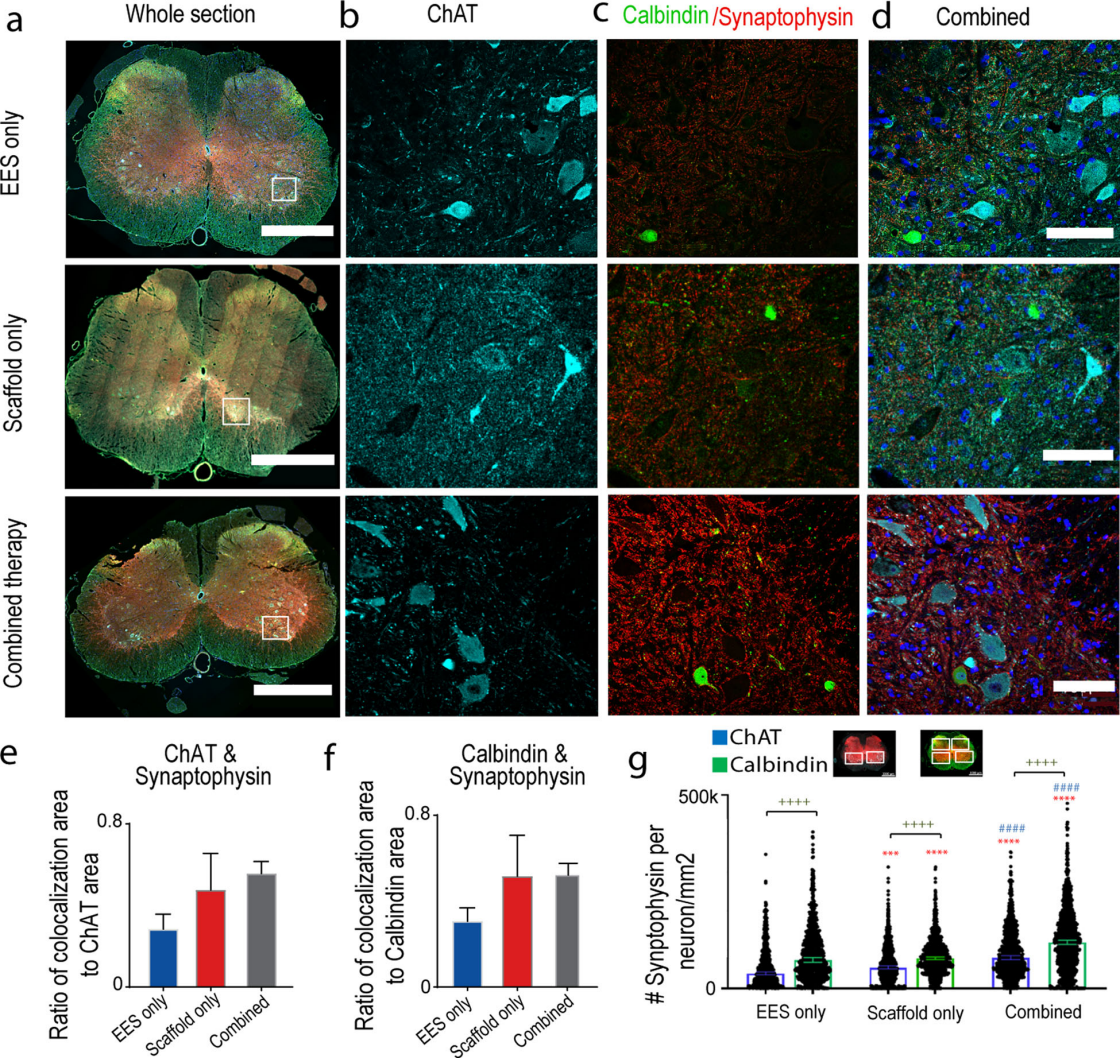
Morphological reorganization of spinal cord below the injury was observed in rats receiving Combined therapy. Changes in synaptic boutons co-localization with motor neurons and interneurons in the lumbosacral spinal cord of rats with EES only, with Scaffold only and with Combined therapy. **a** Image of a transverse section (10 µm thick) of the whole spinal cord taken at 20× (scale bar = 1000 μm) and tiled between spinal segments L2-S1. The spinal cords were immunostained for, **b** choline acetyltransferase (ChAT) positive motor neurons (cyan), **c** calbindin positive interneurons (green) and synaptophysin (red) (scale bar = 100 μm). **d** Combined image with all immunostains and DAPI nuclear staining (blue). **e** Average area of ChAT co-localized with synaptophysin in rats with EES only (*n* = 3), with Scaffold only EES (*n* = 3), and with Combined therapy (*n* = 5). Error bars: +/− standard error of the mean (SEM). **f** Similar comparison for Calbindin co-localized with synaptophysin (not significant on two-way ANOVA with Tukey’s multiple comparisons. Comparison of EES only and combined therapy separately on *t*-test is significant *p* < 0.05). Error bars: +/− standard error of the mean (SEM). **g** Distribution of synaptophysin on ChAT+ motor neuron (EES only: *n* = 913, Scaffold only: *n* = 812, combined therapy *n* = 1086 neurons) and Calbindin+ interneuron (EES only: *n* = 902, Scaffold only: *n* = 954, combined therapy *n* = 1315 neurons) cell bodies per cell area. The bars are shown as median +/− confidence interval. Statistical comparisons were made using Kruskal–Wallis test with Dunn’s multiple comparison (* compared to EES only, # compared to Scaffold Only, + comparison between ChAT and Calbindin; *p < 0.05, **p < 0.01, ***p < 0.001, ****p < 0.0001).

*Morphological reorganization* below the injury was evaluated with synaptophysin co-localized to ChAT positive motor neurons and calbindin positive interneurons (spinal segments L2-S1) (Fig. 5a–d). Rats with combined therapy (0.46 ± 0.088) trended towards greater ratio of co-localization area of ChAT and synaptophysin compared to EES only group (0.28 ± 0.07; Fig. 5e). There was also a trend towards greater synaptophysin co-localized to calbindin in rats with combined therapy (0.45 ± 0.07) compared to EES only (0.31 ± 0.062; Fig. 5f). When the distribution of synaptophysin boutons was quantified on ChAT+ neuronal cell bodies, the EES only group had significantly lower numbers (38,615 synaptophysin boutons per ChAT+ neuron/mm^2^, mean: 54,486,CI_L_: 50,993 CI_U_: 57,979) compared to the animals from the combined therapy group (78,656, mean: 93,287, CI_L_: 89,534 CI_U_: 97,039; *p* < 0.0001) and with scaffold only (53,387, mean: 66,105, CI_L_: 60,893 CI_U_: 71,317; *p* < 0.001). The amount of synaptophysin boutons on ChAT+ neuronal cell bodies was greater in rats with combined therapy compared to scaffold only (*p* < 0.0001; Fig. 5g). Similar difference was found in the distribution of synaptophysin boutons on calbindin+ cell bodies (Fig. 5g). The amount of synaptophysin was greater in rats with combined therapy (118,512 synaptophysin boutons per calbindin+ neuron/mm^2^, mean: 127,661, CI_L_: 122,482 CI_U_: 132,839; *p* < 0.0001) compared to EES only (73,194, mean: 90,682, CI_L_: 85,918 CI_U_: 95,445). The amount of synaptophysin boutons in rats with scaffold only (77,738, mean: 92,142, CI_L_: 85,732 CI_U_: 98,551) was less than the combined therapy groups (*p* < 0.0001), but was not different from EES only. These results indicate that the regenerating axons affect synaptic reorganization below SCI, although, the combined therapy has greater influence on reorganization on the motor and interneuron cells bodies. The synaptophysin distribution was greater on calbindin+ interneurons than the ChAT+ motor neurons (*p* < 0.0001), suggesting the greater role of synaptic reorganization on interneurons with the ratio of synaptophysin per neuron/mm^2^ on calbindin+ to ChAT+ greater for the EES only group (1.89) than the scaffold only (1.46) or combined therapy group (1.51). The synaptic reorganization within the motor neurons (ChAT+ to calbindin+ ratio) was greater for the scaffold only (0.68) and combined therapy (0.66) than the EES only group (0.53).

## Discussion

The results of this study demonstrate the effect of newly regenerated axons on motor performance facilitated with EES through synaptic reorganization of sub-lesional circuitry, suggesting that neuroregenerative and neuromodulatory therapies cumulatively improve motor function after complete SCI. Reconnection across complete transection has been previously achieved with a nerve autograft and scaffolds^74,75^, particularly when delivering cell types and growth factors^57,75–80^. The functional outcome in our study was better in rats who received combined therapy, at the same time, the number of axons through scaffolds was not different between the groups and was in between previously reported with scaffold loaded GDNF secreting Schwann cells^57^ or loaded with rapamycin and Schwann cells^58^. Rapamycin in fact may reduce the number of regenerating axons, although several previous studies demonstrated functional and anatomical improvement when compared with OPF+ scaffolds loaded with Schwann cells alone^39^, which could be attributed to altering synaptic excitability^81–84^. Rapamycin can also block mTOR required for axon regeneration^85,86^, although, its impact on behavior is not evident^61,87–89^. In this study the BBB score at 6 weeks with combined therapy (6.62 ± 1.38) was greater compared to OPF+ with rapamycin microspheres and Schwann cells (4.69 ± 0.57) reported in previous study^39^. BBB score at week 4 in group with combined therapy (4.33 ± 1.31) in this study was similar to effect of OPF+ scaffolds with GDNF Schwann cells (3.67 ± 0.4) reported previously^38^.

Considering limitations of BBB^90,91^ we added electrophysiological and kinematic analysis accommodating our recently developed Multifactorial Behavioral Assessment system^92^. The BBB scale depends on subjective observations of basic hindlimb function on a 21-point scale that may lack sensitivity for some subtle locomotor changes and electrophysiological outcomes of activity^90,93–96^. Measures such as kinematics and EMG provide information on motor recovery, combining multiple sensitive parameters^91,92,97,98^. The Multifactorial Behavioral Assessment system used in this study simultaneously recorded limb and joint kinematics and electrophysiological measurements, such as EMGs and fMEPs^92^. In addition, the body weight support system and treadmill stepping help to record subtle changes in locomotion not possible with traditional BBB testing. Using this assessment, we found improvements in group with combined therapy vs. EES only, and with intermediate effect in scaffolds only group. Following re-transection, group with combined therapy still demonstrated better performance compared to EES only group with complete transection. The difference between EES only vs. combined (scaffold + EES) therapy group indicates on the role of newly regenerating axons in reorganization of sub-lesional circuitry. Electrophysiological changes tested with stimulation of the spinal cord above and below the injury demonstrated two types of functional connectivity with sub-lesional network. The long latency SEPR induced by stimulation of the spinal cord above the injury, likely indicates on activation of polysynaptic network via translesional connectivity and its inhibition after re-transection. Facilitation of the monosynaptic middle response (MR) after re-transections indicates on different mechanism, likely related with pre-synaptic inhibition.

Further observed greater synaptophysin co-localization with motor neurons and interneurons in animals treated with combined therapy suggests that regenerating axons form functional connections with sub-lesional circuitry, facilitating reorganization with EES-enabled training. Synaptic reorganization below the SCI has been attributed to various rehabilitation strategies^94,99^, while the quality of movement is largely depends on descending commands and sensory feedback integration on interneuronal populations^6,13,100–103^. Here we demonstrate that newly regenerating axons along with EES-enabled motor training together influence the sub-lesional circuity through synaptic reorganization. This synaptic reorganization strengthens the network, so that even following re-transection there is improved EES-enabled stepping compared to controls. Although the number of rats in this study is relatively small, the concordance of behavioral, electrophysiological, and histological results convincingly demonstrates that greater synaptic reorganization on the interneurons in the presence of regenerated axons is leading to restoration in polysynaptic responses and improving gait, particularly in group with combined treatment.

In summary, in this work a combination of neuroregenerative scaffold technology with neuromodulatory bioelectronics to delivery EES-enabled rehabilitation was validated and tested in rats with complete spinal transection. The results of this study demonstrate that newly regenerated axons through the cell-containing scaffold with EES-enabled motor training can reorganize sub-lesional circuitry and improve EES-enabled motor performance, providing a platform for synergistic testing and translation of regenerative and neuromodulatory therapies to maximize functional restoration after SCI.

## Methods

### Animal groups

Seventeen Adult female rats (Sprague–Dawley, 250–300 g body weight) were used in this study. All rats were implanted with stimulating electrodes epidurally at T8 and S1 spinal cord levels and EMG electrodes implanted in the hind limb muscles tibialis anterior (TA) and medial gastrocnemius (MG). The groups studied include, (1) rats implanted with EES electrodes but no Scaffold and received EES (EES only; *n* = 4), (2) rats implanted with EES electrodes and scaffold but received no EES (scaffold only; *n* = 3) and (3) rats implanted with EES electrodes and scaffold and received EES-enabled motor training (combined therapy; *n* = 10). In order to study the functional effect of fibers regenerated across the scaffold, group 3 animals were re-transected 6 weeks after complete SCI at level T9.

### Surgical procedures

The rats were deeply anesthetized by a combination of ketamine (100 mg/kg) and Xylazine (10 mg/kg) administered intraperitoneally (IP) and maintained at a surgical level with supplemental doses of ketamine as needed. Buprenorphine was administered as a single dose at the beginning of the experiment. All surgeries were performed under aseptic conditions. All procedures involving animals were approved by the Mayo Clinic Institutional Animal Care and Use Committee and all guidelines were followed in accordance with the National Institute of Health as well as Institute for Laboratory Animal Research and the United States Public Health Services Policy on the Humane Care and Use of Laboratory Animals.

### Electromyography wire and electrode implants for SCI rats

A small skin incision was made at the midline of the skull. The muscles and fascia were retracted laterally, and the skull was thoroughly dried. A 12-pin Omnetics circular connector (Omnetics, Minneapolis, MN) and 12 Teflon-coated stainless-steel wires (AS632, Cooner Wire, CA) were attached to the skull with screws and dental cement as previously described^10,73,104^. Skin and fascia incisions were made to expose the bellies of the MG and TA muscles bilaterally. Using hemostats, the EMG wires were routed subcutaneously from the back incision to the appropriate locations in the hind-limb. Bipolar intramuscular EMG electrodes were inserted into the muscles as described previously^73,104^. The EMG wires were coiled near each implant site to provide stress relief. Electrical stimulation through the head-plug was used to visually verify the proper response of the electrodes in each muscle^73^. A partial laminectomy was then performed at the L2 vertebral level (S1 spinal segment) and one wire was affixed to the dura at the midline using 9.0 sutures as previously described^10^. A small notch made in the Teflon coating (about 0.5–1.0 mm) of the wire used for EES was placed toward the spinal cord and served as the stimulating electrode. Another laminectomy was performed to expose the area for implantation of the electrode at T8 spinal segment. The wire was coiled in the back region to provide stress relief. Teflon coating (about 1 cm) was stripped from the distal centimeter of one wire that was inserted subcutaneously in the back region and served as a common ground^12,73^.

### Poly-lactic-co-glycolic acid (PLGA)-rapamycin microsphere fabrication

Microspheres fabricated from PLGA were used to slowly elute the antifibrotic drug rapamycin. The polymer used to form the PLGA microspheres was 50:50 lactic acid to glycolic acid with 29 kDA molecular weight (Resomer RG 503 H; Sigma-Aldrich). An oil-in-water emulsion with solvent evaporation technique as previous described^39,105,106^. Briefly, 1 mg of rapamycin was dissolved in 100 µL of absolute ethanol. The rapamycin-ethanol solution vortex emulsified dropwise for 30 s in 250 mg PLGA dissolved in 1 mL of methylene chloride. The mixture was then emulsified in 2 mL of 2% (w/v) poly vinyl alcohol for 30 s. This solution was then mixed with 100 mL of 0.3% (w/v) poly vinyl alcohol and 100 mL of 2% (w/v) isopropanol and stirred for 1 h to evaporate methylene chloride. The microspheres are then centrifuged at 2000 rpm for 3 min and washed three times with distilled water and centrifugation. The liquid is then discarded, and the microspheres are frozen at −80 °C for 1 h. Lastly, the microspheres are dried overnight under vacuum and then used for scaffold manufacture.

### Scaffold preparation

Positively charged OPF+ scaffolds with seven channels (Fig. 1a; and Supplementary Fig. 2) were fabricated as previously described^39,107,108^. Briefly, the liquid polymer consists of 1 g of OPF macromere (16,246 g/mol) dissolved in 650 µL of deionized water, 0.05% (w/w) photoinitiator (Irgacure 2959; Ciba Specialty Chemicals) 0.3 g N-vinyl pyrrolidinone (NVP; Sigma), and 20% [2-(methacryloyloxy) ethyl]-trimethylammonium chloride (MAETAC; Sigma). To embed the rapamycin microspheres, 25 mg of microspheres was stirred into 250 µL of OPF+ polymer liquid. The liquid polymer containing the microspheres is then mold injected over seven equally spaced wires with 290 µm diameter in a glass cylinder. After 1 h exposure the UV light (365 nm) ay 8 mW/cm^2^, the individual scaffolds are cut into 2 mm lengths. Before use, they are serially washed in ethanol three times for 30 min each followed by 4 1× PBS washes^39^.

The characteristics of this scaffold have been described in earlier studies^27,39,58^. Briefly, the OPF+ hydrogel has a compression moduli of 0.13 ± 0.03 MPa and flexural moduli of 1.87 ± 1.03 MPa, which was similar to the rat spinal cord compression moduli of 0.19 ± 0.09 MPa and flexural moduli of 0.74 ± 0.14 MPa^27^. There is a weight loss of 14% for OPF+ in PBS after 5 weeks, showing a slow degradation rate due to the highly crosslinked structure^58^. The PGLA microspheres were found to be of a mean diameter of 57.42 ± 17.63 μm^58^. The encapsulation efficiency of rapamycin was 100 ± 10% for a loading dose of 1 mg/250 mg PLGA^39^. The release kinetics in vitro of rapamycin from PLGA microspheres were found to be a burst release in the first week (approximately 25%), following by a slower release over the second week (approximately 10%), and steady release of rest of the contents over the next 3 weeks^39^.

### Primary and GDNF-secreting Schwann cells

Glial derived neurotrophic factor (GDNF, accession: NM_000514) secreting SCs were created as described previously^57^. Briefly, primary rat SCs were harvested from the sciatic nerve of Sprague Dawley pups between postnatal day 2 and 5. The nerves are stripped of connective tissue and the epineurium and cut into 1 mm sections. Then are enzymatically treated with 0.25% trypsin EDTA (Mediatech Inc.) and 0.03% collagenase (Sigma). The cells are mechanically dissociated by pipetting and centrifuges for 5 min at 188G. The primary SCs were then genetically modified by seeding 80,000 cells/mL and grown with high titer (2 × 1010 cfu) retroviral (GDNF-eGFP) supernatant media with 8 µg/mL polybrene for 24 h. Transduced SCs were selected for 12 days with 1 mg/mL G418 analog in SC media containing 50:50 DMEM: F12 media (GIBCO) supplemented with 10% FBS, 1% antibiotic–antimycotic, 2 µM Froskilin (Sigma), and 10 ng/mL neuregulin-1 (recombinant human NRG-1; R&D Systems)) at 37 °C in 5% CO_2_. The cells were expanded and frozen until ready for use.

Sterile RAPA-OPF+ scaffolds were loaded with GDNF-SC at a density of 100,000 cells/μL suspended in Matrigel (BD Biosciences) using gel loading pipette tips as previous described^38^. The internal volume of channels the scaffolds are 0.67 μL, therefore the total amount of cells loaded into the scaffold were 476,000 cells. The loaded scaffolds were incubated for 48 h at 37 °C in 5% CO_2_ before implantation into animals.

### Spinal cord transection and scaffold implantation

One week after electrode implantation surgery, a complete spinal cord transection was performed. Rats were anaesthetized with a mixture of oxygen and Isoflurane (≈1.5%). Mid-dorsal skin incision was made between T6 and L4 and the paravertebral muscles were retracted as needed. A partial laminectomy was performed at the T9 level and the dura was opened longitudinally. Lidocaine was applied locally and the spinal cord was completely transected using a microscissors. Completeness of the lesion was verified by two surgeons under microscope. The spinal cord retracted as a result of the transection resulting in a 2 mm gap (Supplementary Fig. 2D). Then, some rats were implanted with 2 mm GDNF/SC-RAPA-OPF+ scaffold in the space created by transection (Supplementary Fig. 2E) with the channels aligned with the rostral and caudal stumps as previously described^27^. Tight contact in between the stumps were confirmed through observation under a surgical microscope. The muscle was sutured with a deep tight first suture over the scaffold in order to hold it in place. Buprenorphine (0.5–1.0 mg/kg subcutaneous injections twice a day) were administered for analgesia. Tissues were sutured by layers and animals were allowed to recover in individual cages with soft bedding. Manual bladder expression was performed four times daily for two weeks post transection. Similar to our previous studies with complete SCI, about 2 weeks after injury rats have regained bladder function and then were monitored twice daily 5 days a week for the duration of the study. The rats that demonstrated delayed in bladder functions recovery were continued to be manually expressed 4 times daily until the function has recovered. The rats were allowed to move over an open field surface to help stimulate their bladder and reflexes once daily for 2 weeks. The hind limbs of the spinal rats were also moved passively through a full range of motion once per day to maintain joint mobility.

### Spinal cord electrical epidural stimulation

A single channel manually controllable isolated stimulator (A-M systems, Sequim, WA) or an eight independent channel real-time programmable (STG4008, Multichannel Systems, Reutlingen) stimulator were used to deliver biphasic square wave electrical stimulation (250 μs pulse width) at 40 Hz with amplitudes ranging from 0.5 to 2.5 V to the epidural electrode placed on the rat’s lumbosacral (S1) spinal cord to facilitate motor activity.

### Training and animal care

Rats were acclimated to a specially designed motor-driven rodent treadmill and body weight support system^92^ for a period of 7 days prior to surgery for 15–20 min each day (Fig. 1b). One week post-surgery, the rats went through a manual bipedal step training rehabilitation process (30 min a day, 3 days a week) for 6 weeks under the influence of EES at 40 Hz subthreshold level (0.4–2 V). Chronic step training was used because it helps to engage and reinforce the locomotor networks.

### Basso, Bettie, and Bresnahan (BBB) assessment

The BBB locomotor rating scale was used to access the hind-limb function weekly starting one-week post-injury (Fig. 1c). Three independent observers were blinded to the animal groups and score was given on the 21-point scale. The BBB scoring was performed as previously described^93^, except that the animals were scored twice with and without subthreshold EES (40 Hz). At least 30 min of rest was given between the sessions. The BBB scores from the independent observers were averaged and then the left and right limb scores were averaged for each rat. Two-way ANOVA with Tukey’s multiple comparisons was used to determine statistical difference between groups.

### Kinematics

A motion tracking system (Vicon, UK) was used to record three-dimensional digital position of back and the hind limb joints at 100 Hz. Six motion-sensitive Infrared (IR) cameras were aimed at the treadmill or the open field volumes. Another high-speed video camera synchronized with motion tracking system was positioned in a side to provide a lateral view of the motor performance. Retro-reflective markers were placed on bony landmarks at the iliac crest, greater trochanter, lateral condyle of the femur, lateral malleolus and the distal end of the fifth metatarsal on both legs of the rat to record the kinematics of the hip, knee, and ankle joints. Nexus system was used to obtain three-dimensional coordinates of the markers. Analysis of kinematic data was performed using method previously described^92^.

### Electrophysiology

EMG activity was collected from TA and MG muscles on both legs at 4000 Hz during stepping and later high-pass filtered at 0.5 Hz to remove direct current offset. In order to determine flexor (TA) and extensor (MG) coordination, EMG signals were band pass filtered (10–1000 Hz), rectified, normalized and plotted on *X* and *Y* axis consecutively^92^. The excitability of the spinal cord were determined by stimulating the spinal cord at S1 spinal level while *SCMEP* were recorded from the hind-limb muscles via EMG^73^, while the rats were suspended in the body weight support system^92^. The functional connectivity across the injury and reflexes below the injury were tested by stimulating T8 spinal level above the injury, while recording SEPR from hindlimb muscles. During testing, the rats were suspended allowing hind limbs to freely respond and stimuli of increasing intensity (amplitude 0.5–4.5 V, pulse width 0.25 ms, frequency 0.5 Hz) were applied to epidural spinal electrodes. Ten consecutive responses were collected during spinal cord stimulation above (T8) and below (S1) the injury with a single pulse to evaluate SEPR and SCMEP in awake animals to assess the influence of information delivered across scaffold during standing and during stepping on a treadmill.

### Perfusion and dissection

Seven weeks of post-injury that rats were transcardially perfused using 4% paraformaldehyde (PFA) in phosphate buffered saline (PBS). The spinal column was removed en block and post-fixed in 4% PFA in PBS for 2 days at 4 °C. The spinal columns were then washed three times in PBS and dissected out. Then 6 mm segment containing the scaffold at the center and adjacent spinal cord was embedded in paraffin. A 1 cm block covering L2-S1 spinal cord segment was also embedded in paraffin. Transverse or longitudinal serial sections of 10 μm thick were made on a Reichert-Jung 23 Biocut microtome (Leica, Bannockburn, IL) of the scaffold and spinal cord areas.

### Immunohistochemistry

Post-mortem immunohistochemistry was performed in all groups of animals in order to assess regeneration through the scaffold and plasticity change in lumbosacral spinal cord (Fig. 1d). Primary antibodies used were against β-III tubulin (Tuj-1; mouse anti-rat, 1:300; Millipore), choline acetyltransferase (ChAT; goat anti-rat. 1:50, Millipore), calbindin (CB38; rabbit, 1:1000, SWANT), and synaptophysin (mouse, 1:50, Abcam). The secondary antibodies used were against mouse Cy3 (donkey, 1:200, Jackson ImmunoResearch Laboratories), goat Alexa 647 (donkey, 1:200, Jackson ImmunoResearch Laboratories), rabbit Cy2 (donkey, 1:200, Jackson ImmunoResearch Laboratories). The slides were deparaffinized through serial washes through xylene, ethanol, and distilled water. Antigen retrieval was performed through incubation in 1 mM EDTA in PBS for 30 min in a rice steamer. The sections were then blocked using 10% normal donkey serum in 0.3% Triton X-100 in PBS for 1 h. Next, TrueBlack (Biotium) was used as per manufacturer’s instructions to quench autofluorescence due to lipofuscin. Then, the sections were incubated overnight in the primary antibody diluted in 5% normal donkey serum in PBS at 4 °C. The next day after three washes in PBS, the sections were incubated in secondary antibody diluted in PBS containing 5% normal donkey serum for 1 h at room temperature. Lastly, the sections were washed three times in PBS containing, and then mounted with SlowFade Gold Antifade Reagent with DAPI (Molecular Probes, Eugene, Oregon, USA).

### Axon count

Axons were identified as punctate staining of β-III tubulin and were counted in transverse paraffin sections at quarter lengths throughout the scaffold. Tiled 20× images of the whole scaffold were imaged using a Zeiss LSM 780 inverted confocal microscope. StereoInvestigator software suite (MBF) was used to count the axons in each channel using Optical Fraction Probe. Briefly, a square grid measuring 100 µm × 100 µm was overlaid on the image after contouring around the channel. The axon was counted if it fell either in the quadrant frame or overlapped with the acceptance lines (top horizontal and right vertical boundary). The average axon counts per channel for each quarter length were averaged yielding one value per animal. The total axon counts of rats with scaffolds and with/without EES were compared using two sample *t*-test.

### Colocalization of synaptophysin with neuronal populations

Antibody against ChAT was used to identify motor neuron populations, while Calbindin was used to identify interneuron subtype on sections taken from transverse and longitudinal spinal cords between spinal segments L2-S1. The co-localization tool in Image-Pro (Media Cybernetics) was used to determine the amount of overlap of synaptophysin with ChAT or Calbindin according to manufacturer’s instructions. The synaptophysin labeled presynaptic boutons interacting with these cell types. Color pairs were created between the red (synaptophysin) and green (Calbindin) channels and the red and far red channels (ChAT). A threshold was applied using the auto-bright feature. The process was standardized and automated to have consistent, unbiased analysis. The analysis was done using 40× images taken on a Zeiss LSM 780 inverted confocal microscope serially through the lumbosacral spinal cord every 1500 µm. On each slide two sections 20 µm apart were chosen and on each section four areas were chosen and titled: the left and right dorsal horn and ventral horn (Supplementary Fig. 3A). The areas of interest were then averaged per section, the two sections per slide were averaged, and then all the slides were averaged to yield one average per animal. The amount of co-localization was compared between animals with scaffolds and EES that were re-transected, scaffold only, and no scaffold and EES using a one-way ANOVA with Tukey’s multiple comparisons.

Distribution of synaptophysin boutons on ChAT+ and calbindin+ cell bodies was analyzed using a semiautomated image analysis system on the QuPATH open source software^109^ with the images described above. The cell bodies labeled with ChAT or calbindin were first traced (Supplementary Fig. 3B). The cell area of the traced space was recorded. Then the cell detection tool was used to identify the synaptophysin boutons. The detection channel was set for the red channel and then optimized parameters were used (requested pixel size 0 µm, background radius 0 µm, median filter radius 1 µm, sigma 0.5 µm, minimal area 0.01 µm^2^, maximum area 5 µm^2^, threshold 30). The number of synaptophysin boutons per neuron were normalized to cell body area and all the neurons were plotted. Then the median with upper 95% confidence interval (CI_U_) and lower 95% confidence interval (CI_L_) was used to indicate the peak of the distribution. Kruskal–Wallis test with Dunn’s multiple comparison was used to analyze the distribution.

### Statistical analysis

Statistical analysis was performed using SigmaPlot (Systat Software, UK) or Prism (GraphPad). The data were first tested for normality and then one-way Analysis of Variance (ANOVA) was performed to determine if the groups had different outcomes. If the groups were found significantly different (*p* < 0.05) a post-hoc analysis was performed using Holm-Sidak method (unless otherwise stated) to compare against control and *p* values were reported. When compared between two groups, two samples *t*-test was performed. All results were reported as mean ± standard error, and ^*,#,$^*p* < 0.05, ^**,##,$$^*p* < 0.01, and ^***,###,$$$^*p* < 0.001, unless otherwise noted. Nonparametric analysis was done using Kruskal–Wallis test with Dunn’s multiple comparison. Two-way ANOVA with Tukey’s multiple comparisons was done where there were multiple groups and conditions.

### Reporting summary

Further information on research design is available in the Nature Research Reporting Summary linked to this article.

## Supplementary information

Supplementary Information

Reporting Summary

## Data Availability

All data is available on reasonable request.
