# Supplementary material for: Newly regenerated axons via scaffolds promote sub-lesional reorganization and motor recovery with epidural electrical stimulation
Source: NPJ Regen Med. 2021 Oct 20;6:66. doi: 10.1038/s41536-021-00176-6 (PMC8528837; doi:10.1038/s41536-021-00176-6)
Supplement: Supplementary file 1 — Supplementary Information [file 41536_2021_176_MOESM1_ESM.pdf]

Supplementary Notes:

Glossary

Basso, Beattie, Bresnahan (BBB) locomotor scale: A limb motor skills assessment used to measure recovery after spinal cord injury in rats over a 21-point observational scale.

Epidural Electrical Stimulation (EES): Application of electrical current to the dorsal dural surface of the spinal cord.

Kinematics: Measurement of different movements used to describe gait and motion.

Middle Response (MR): An EMG response to the spinal cord stimulation primary related to monosynaptic connectivity.

Metatarsophalangeal (MTP) joint: The joint between the proximal bones of the toes and the metatarsal bones of the foot.

Neuromodulation: The regulation of neuronal activity by electrical or other modalities of stimulation.

Neuroregeneration: Regrowth and repair of nervous tissue.

Sub-lesional: Refers to the changes or circuitry below the lesion.

Subfunctional connections: Refers to the stated where clinically absent connectivity could be facilitated to the functional connectivity. For example, subfunctional circuitry in this study is silent without training and outside stimulation.

Supra-lesional: Refers to the changes or circuitry above the lesion

Supralesion Evoked Polysynaptic Response (SEPR): Polysynaptic response induced by stimulation of the spinal cord above the injury.

Trans-lesional: Across the lesion

Supplementary Figures:

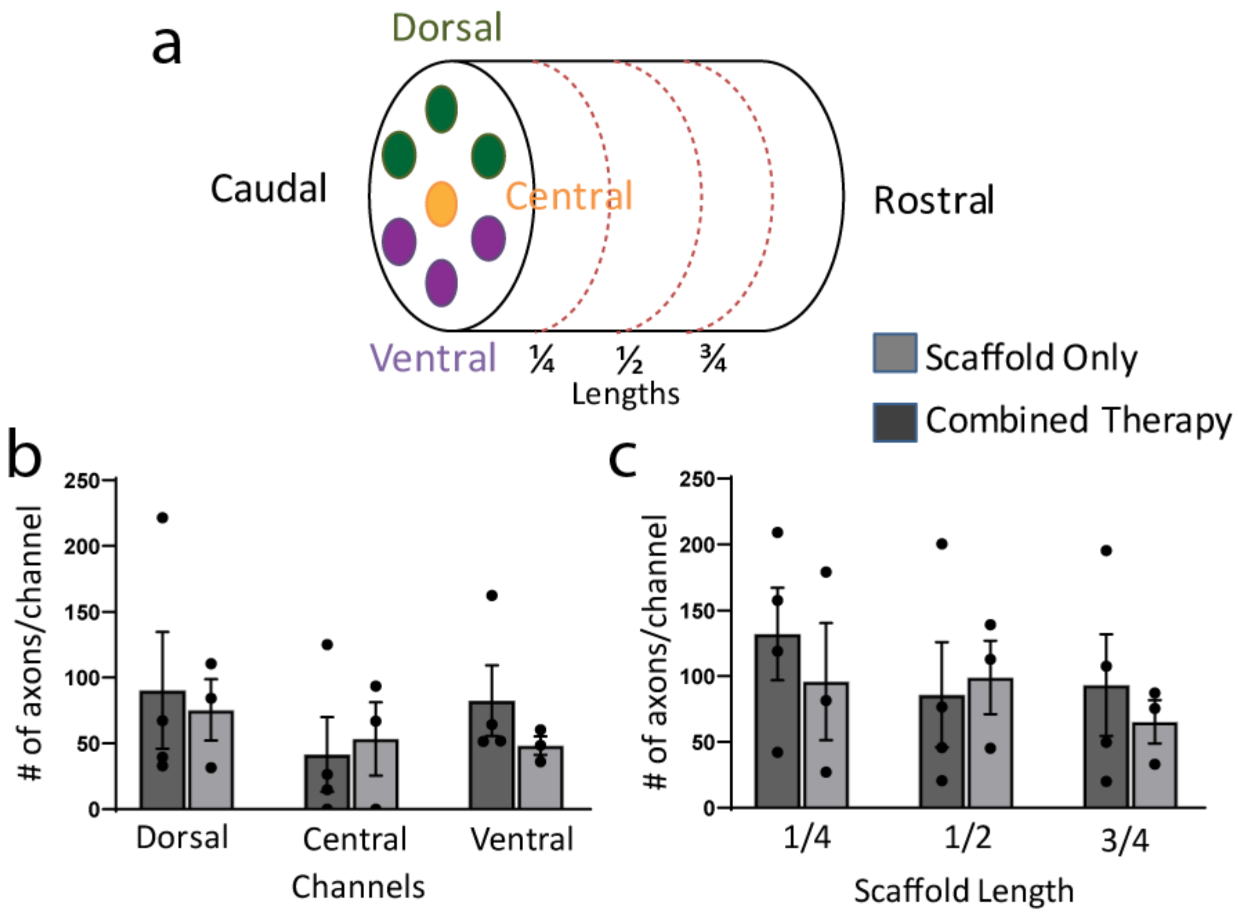

Supplementary Figure 1: Axon counts through different segments of the scaffold. (a) Scaffold sectioning method applied in the experiment. Each scaffold was sectioned at ¼, ½ and ¾ lengths and the number of axons at each channel was counted in all three sections. The three top channels on the dorsal side are identified as dorsal (green), the central channel (orange) and the three channels on the ventral side is identified as ventral (purple). (b) Number of axons regenerated per channel in the dorsal, central, and ventral channels are compared between the rats with scaffold only and rats receiving combined therapy. (c) Similar comparison of number of axons regenerated per channel at ¼, ½ and ¾ lengths of the scaffold.

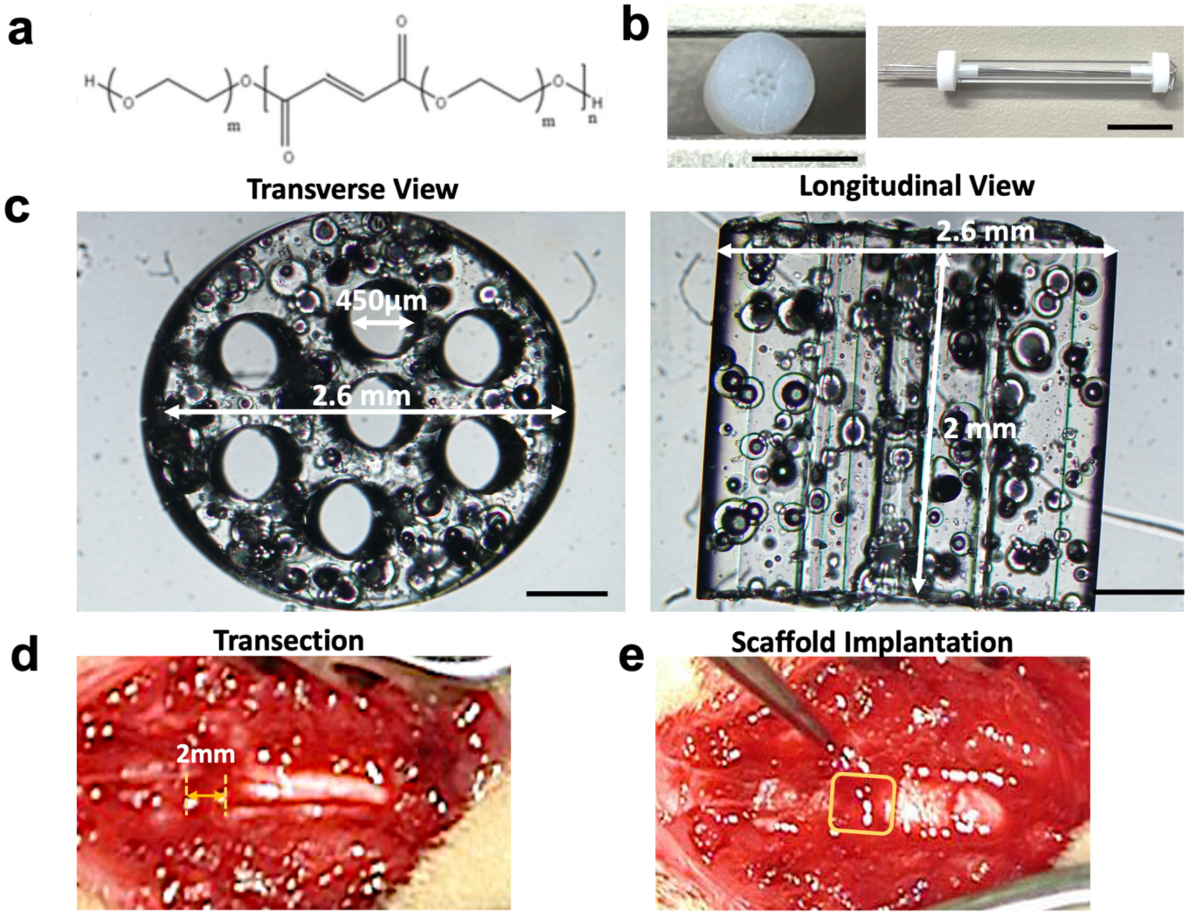

Supplementary Figure 2: Fabrication of positively charged oligo[poly(ethylene glycol) fumarate] (OPF+). (a) The chemical structure of OPF+. OPF+ is synthesized from 1g of OPF powder dissolved in 650 µL of deionized water, 0.05% (w/w) of photoinitiator (Irgacure 2959), and 0.3 g of N-vinyl pyrrolidinone. (b) The liquid OPF+ polymer with embedded rapamycin microspheres are injected into a glass mold (right) with wires running parallel in a multichannel shape though 2 Teflon plugs (left) and crosslinked using UV (right scale bar = 5 mm, left scale bar = 1 cm). (c) The resulting scaffold when hydrated is 2.5 mm in diameter with 450 um diameter channels (left, modified from 58). The scaffold is 2 mm thick (right) (scale bar = 500 µm). (d) A transection of the thoracic level 9 results in a natural 2 mm gap. (E) The scaffold is placed within the transection site and the muscles are sutured with a tight suture over the scaffold.

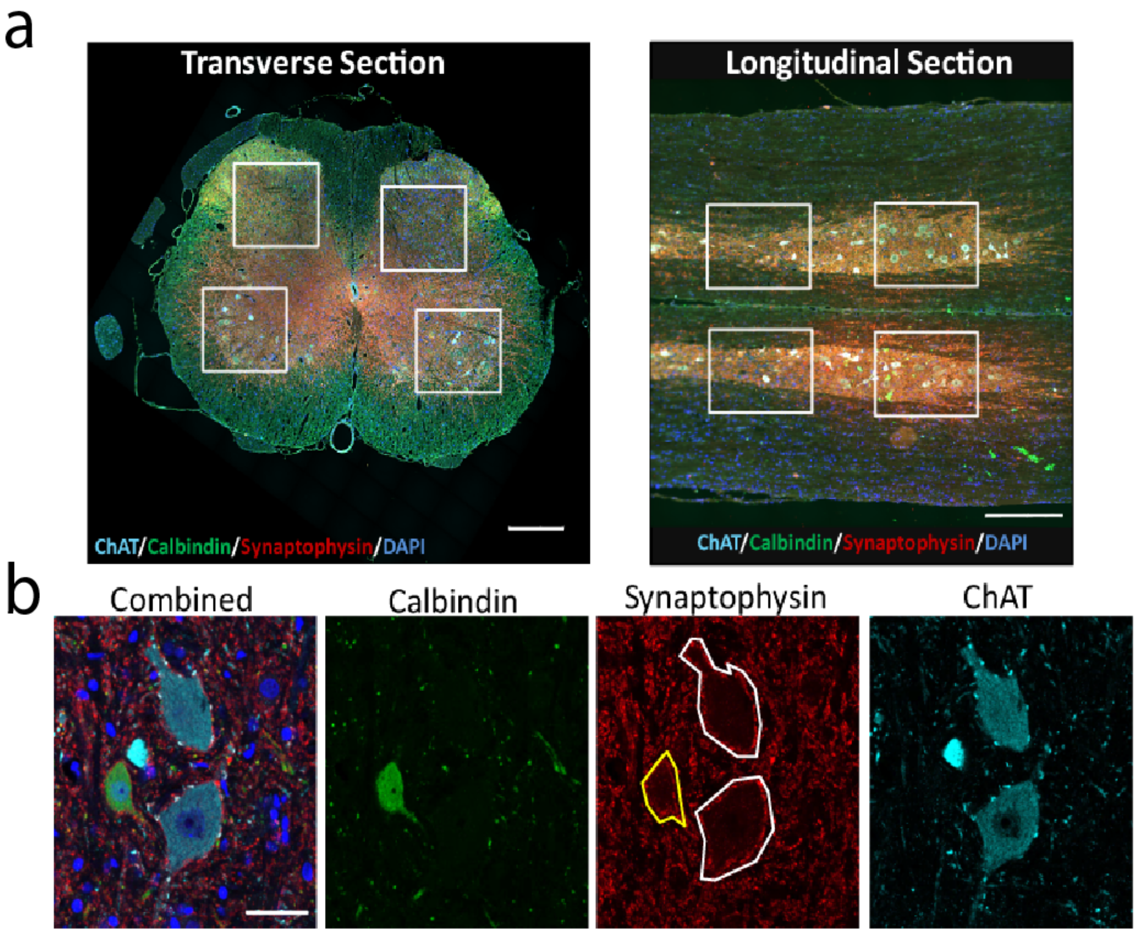

Supplementary Figure 3: Methodology of synaptophysin colocalization and bouton counts with ChAT and calbindin. (a) Representative examples of one transverse and longitudinal section of lumbosacral spinal cord used in the analysis. The spinal cord sections are co-stained with ChAT, Calbindin, Synpatophysin and DAPI. Four regions (enclosed by white squares) in the dorsal and ventral regions were selected to perform analysis on plasticity change due to regeneration through the scaffold (scale bar = 500 µm). (b) Representative example of immunohistochemistry performed on the lumbosacral spinal cord, combined DAPI, Calbindin, Synaptophysin, ChAT and individual stained images shown at 40x zoom (scale bar = 50 µm). The area around the cell body were traced (white tracing for ChAT positive cells and yellow for calbindin positive cells) to analyze the amount of synaptophysin in the cell boundary.
