# Supplementary material for: Newly regenerated axons via scaffolds promote sub-lesional reorganization and motor recovery with epidural electrical stimulation
Source: NPJ Regen Med. 2021 Oct 20;6:66. doi: 10.1038/s41536-021-00176-6 (PMC8528837; doi:10.1038/s41536-021-00176-6)
Supplement: Supplementary file 2 — Reporting Summary [file 41536_2021_176_MOESM2_ESM.pdf]

nature research

Corresponding author(s): Igor Lavrov

Last updated by author(s): Aug 24, 2021

Reporting Summary

Nature Research wishes to improve the reproducibility of the work that we publish. This form provides structure for consistency and transparency in reporting. For further information on Nature Research policies, see our Editorial Policies and the Editorial Policy Checklist.

Statistics

For all statistical analyses, confirm that the following items are present in the figure legend, table legend, main text, or Methods section.

n/a	Confirmed	
☐	☒	The exact sample size (*n*) for each experimental group/condition, given as a discrete number and unit of measurement
☐	☒	A statement on whether measurements were taken from distinct samples or whether the same sample was measured repeatedly
☐	☒	The statistical test(s) used AND whether they are one- or two-sided<br>*Only common tests should be described solely by name; describe more complex techniques in the Methods section.*
☐	☒	A description of all covariates tested
☐	☒	A description of any assumptions or corrections, such as tests of normality and adjustment for multiple comparisons
☐	☒	A full description of the statistical parameters including central tendency (e.g. means) or other basic estimates (e.g. regression coefficient) AND variation (e.g. standard deviation) or associated estimates of uncertainty (e.g. confidence intervals)
☐	☒	For null hypothesis testing, the test statistic (e.g. *F*, *t*, *r*) with confidence intervals, effect sizes, degrees of freedom and *P* value noted<br>*Give P values as exact values whenever suitable.*
☒	☐	For Bayesian analysis, information on the choice of priors and Markov chain Monte Carlo settings
☒	☐	For hierarchical and complex designs, identification of the appropriate level for tests and full reporting of outcomes
☐	☒	Estimates of effect sizes (e.g. Cohen's *d*, Pearson's *r*), indicating how they were calculated

Our web collection on statistics for biologists contains articles on many of the points above.

Software and code

Policy information about availability of computer code

Data collection	A single channel manually controllable isolated stimulator (A-M systems, Sequim, WA) or an eight independent channel real-time programmable (STG4008, Multichannel Systems, Reutlingen) stimulator were used to deliver biphasic square wave electrical stimulation. A motion tracking system (Vicon, UK) was used to record three-dimensional digital position of back and the hind limb joints at 100 Hz.
Data analysis	Statistical analysis was performed using SigmaPlot (Systat Software, UK) or Prism (GraphPad)

For manuscripts utilizing custom algorithms or software that are central to the research but not yet described in published literature, software must be made available to editors and reviewers. We strongly encourage code deposition in a community repository (e.g. GitHub). See the Nature Research guidelines for submitting code & software for further information.

Data

Policy information about availability of data

All manuscripts must include a data availability statement. This statement should provide the following information, where applicable:

- Accession codes, unique identifiers, or web links for publicly available datasets
- A list of figures that have associated raw data
- A description of any restrictions on data availability

All data is available on request

Field-specific reporting

Please select the one below that is the best fit for your research. If you are not sure, read the appropriate sections before making your selection.

☒ Life sciences ☐ Behavioural & social sciences ☐ Ecological, evolutionary & environmental sciences

For a reference copy of the document with all sections, see nature.com/documents/nr-reporting-summary-flat.pdf

Life sciences study design

All studies must disclose on these points even when the disclosure is negative.

Sample size	The necessary and sufficient number in each group was determined using standard power calculations based on the mean ± SD for specific outcome measures
Data exclusions	No data were excluded
Replication	Within this experimental design all attempts to replication were successful
Randomization	Seventeen Adult female rats were used in this study. The groups included: (1) rats implanted with EES electrodes but no Scaffold and received EES, (2) rats implanted with EES electrodes and scaffold but received no EES and (3) rats implanted with EES electrodes and scaffold and received EES-enabled motor training.
Blinding	Data were analyzed blindly in this study by researchers who did not participate in animal training,

Reporting for specific materials, systems and methods

We require information from authors about some types of materials, experimental systems and methods used in many studies. Here, indicate whether each material, system or method listed is relevant to your study. If you are not sure if a list item applies to your research, read the appropriate section before selecting a response.

Materials & experimental systems

n/a	Involved in the study	
☒	☐	Antibodies
☒	☐	Eukaryotic cell lines
☒	☐	Palaeontology and archaeology
☐	☒	Animals and other organisms
☒	☐	Human research participants
☒	☐	Clinical data
☒	☐	Dual use research of concern

Methods

n/a	Involved in the study	
☒	☐	ChIP-seq
☒	☐	Flow cytometry
☒	☐	MRI-based neuroimaging

Animals and other organisms

Policy information about studies involving animals; ARRIVE guidelines recommended for reporting animal research

Laboratory animals	Seventeen Adult female rats (Sprague–Dawley, 250–300 g body weight)
Wild animals	N/A
Field-collected samples	N/A
Ethics oversight	All procedures involving animals were approved by the Mayo Clinic Institutional Animal Care and Use Committee and all guidelines were followed in accordance with the National Institute of Health as well as Institute for Laboratory Animal Research and the United States Public Health Services Policy on the Humane Care and Use of Laboratory Animals

Note that full information on the approval of the study protocol must also be provided in the manuscript.
